# Onabotulinumtoxin A Improves Psychological Aspects in Chronic Migraine Patients

**DOI:** 10.3389/fneur.2020.633355

**Published:** 2021-01-27

**Authors:** Jasem Youssef Al-Hashel, Hasan Kh Ashkanani, Ohood Almutairi, Fajer A. Bokubar, Shahad Mubarak, Sawsan Alwazzan, Raed Alroughani, Doaa Youssry, Samar Farouk Ahmed

**Affiliations:** ^1^Neurology Department, Ibn Sina Hospital, Kuwait, Kuwait; ^2^Faculty of Medicine, Kuwait University, Kuwait, Kuwait; ^3^Division of Neurology, Department of Medicine, Amiri Hospital, Kuwait, Kuwait; ^4^Department of Neurology, Cairo University, Cairo, Egypt; ^5^Neuropsychiatry Department, Faculty of Medicine, Minia University, Minia, Egypt

**Keywords:** Onabotulinumtoxin A, chronic migraine, depression, anxiety, sleep disturbance

## Abstract

**Background:** Chronic migraine (CM) affects 5.4% of the Kuwaiti population. It is associated with significant headache-related disability, psychiatric comorbidity and reduced quality of life. The aim of this study is to assess the efficacy of Onabotulinumtoxin A on psychological aspects of chronic migraine patients.

**Methods:** This prospective study over 36 months included chronic migraine patients in a tertiary headache center. Eligible patients met International Classification of Headache Disorders disorders-third edition, beta version (ICHD-III) revision criteria for chronic migraine. Patients with history of psychiatric or medical problems other than migraine disorders were excluded. Patients who received less than 4 injections cycles of Onabotulinumtoxin A were excluded. Identified patients received 155 units of Onabotulinumtoxin A quarterly according to the Phase III Research Evaluating Migraine Prophylaxis Therapy Trail (PREEMPT) protocol. Quality of life, the seven-item Generalized Anxiety Disorder (GAD-7) scores, the nine-item Patient Health Questionnaire (PHQ9), and the Pittsburgh Sleep Quality Index (PSQI) were collected before injection and at the end of the study. Mean comparison tests were performed using the independent sample *t-*test to assess the effects of Onabotulinumtoxin A on quality of life and comorbid symptoms of anxiety, depression, and quality of sleep.

**Results:** The study identified 131 chronic migraine patients with a mean age of 44.92 years, mean disease duration of 12.20 years and a mean treatment sessions of 7.58. In their last visit, most of our sample showed improvement in quality of life (81%), GAD-7 (81%), PHQ9 (79%), and PSQ1 (76%). The mean score of patient satisfaction was 7.21. Onabotulinumtoxin A treatment for CM improved quality of life significantly (72.92 vs. 103.62*; P* < 0.0001). It was also associated with significant reduction in anxiety [GAD-7 (12.00 vs. 6.61*; P* < 0.0001)] and depression [PHQ-9 (17.91 vs. 12.52; *P* < 0.0001)] scores, as well as reduced difficulty in sleeping [PSQI (12.60 vs. 6.66; *P* < 0.0001)] at the last visit.

**Conclusion:** Prophylactic Onabotulinumtoxin A treatment for CM was associated with significant improvement of quality of life, reduction in symptoms of anxiety and depression, as well as improved symptoms of poor sleep.

## Background

Migraine, a primary type of headache, is a common disabling disorder that can be divided into episodic and chronic migraine (1). The global prevalence of migraine is 14.4%, for females, and 9.8% for males (2). Global studies show that ~1.4–2.2% of the world's population have been diagnosed with chronic migraine (2). Comparatively, the 1-year prevalence of migraine in Kuwait was 23%, which is significantly higher (3). According to the latest Global Burden of Disease Study, headaches, including migraines, ranked second in the leading causes of disability, showing the large burden of the disorder (4). Migraine majorly affects the everyday lives of its sufferers, negatively affecting productivity, and even schooling (5). This burden is also increased by the comorbid psychiatric conditions that occur in association with it, including depression, anxiety, and sleep disorders. Large scale, population-based studies showed that patients with migraine are 2.2–4.0 times more likely to have depression (6). Moreover, in those who have episodic migraines, depression was associated with an increased risk of developing chronic migraine (7). In addition, the diagnosis of generalized anxiety disorder was significantly more prevalent in migraineurs than in those without migraine (8). Generalized anxiety disorder is often comorbid with major depressive disorder (MDD) and both of them increase the burden of migraine (8). Therefore, patients with migraine and psychiatric comorbidities may benefit from preventive therapy to reduce the attacks.

Sleep has also been demonstrated to have a clear relationship with migraine. Chronic migraine patients (CM) reported shorter nightly sleep periods than those with episodic migraine, and they were more likely to exhibit trouble initiating sleep, staying asleep, and sleep triggering headache. Also, complaints of those insomnia symptoms were at least three-fold greater in those patients than the general population (9). This shows the importance of management in migraine patients in the hope of decreasing this socioeconomic and medical burden. Therefore, patients with migraine and psychiatric comorbidities may benefit from preventive therapy to reduce the attacks.

Onabotulinumtoxin A was approved as a prophylactic therapy in adult patients with chronic migraine by the United State Food and Drug Administration (FDA) in 2010 (10). Onabotulinumtoxin A has been proven to reduce not only headache frequency but also the symptoms of depression, anxiety, as well as poor sleep quality in migraine patients (11).

While Onabotulinumtoxin A is known to cause muscle paralysis, the exact mechanism by which it relieves chronic migraine is not clear as of yet. However, some evidence suggests it does so by reducing local nerve sensitization by local inhibition of neuropeptide release, thus resulting in an indirect reduction of central sensitization (12). The neuropeptide calcitonin gene-related peptide (CGRP), which is known to be a major player in migraine, might be involved in regulating sleep maintenance at night (13, 14). Therefore, Onabotulinumtoxin A treatment may help to improve sleep in migraine patients by repressing CGRP from activated sensory neurons and by directly decreasing the amount of CGRP released from trigeminal neurons (15).

Although the socioeconomic burden of migraine in the Kuwaiti population was previously studied (3), no study to date has assessed the psychological aspects as mood and sleep suffering from migraine in patients in Kuwait. Therefore, this study aims to show the effect of treatment on improvement of mood and sleep in chronic migraine patients who received Onabotulinumtoxin A injections.

## Method

This prospective, questionnaire-based study was conducted in a specialized headache clinic in a tertiary hospital in Kuwait. The study started on 1st October 2016 until 30 September 2019. The study population included both male and female patients aged 18–65 years who are diagnosed with CM with or without medication over use headache (MOH). Diagnoses were confirmed by headache specialist according to International Classification of Headache Disorders III (ICHD-III) (1). The study excluded patients with psychiatric disorders or chronic medical problems.

Patients with a diagnosis of another headache disorder, previous use of any Onabotulinumtoxin A for treatment of any headache (at any time) or for any other reason in the last year, pregnant or breast-feeding patients, and patients who did not complete at least three treatment cycles were excluded from the study. In addition, patients who had psychiatric comorbidities, history of previous psychiatric diseases, other migraine comorbidities, such as hypertension, obesity, or smoking, or those taking other medications from Onabotulinumtoxin A for migraine were not eligible for inclusion in the study. Study excluded patients who are on tricyclic antidepressant or selective serotonin reuptake inhibitors as prophylactic treatment for chronic migraine. Also, patients who received <4 injections cycles of Onabotulinumtoxin A were excluded.

Patients were injected intramuscularly with Onabotulinumtoxin A according to the PREEMPT protocol, 155 units divided into 31 injection sites around the head and neck, with sessions occurring in every 3 months (10).

A total of four questionnaires were used to interview patients in order to collect the data for the study. The questionnaires used in the study include The Quality of Life (QOL) (16), Generalized Anxiety Disorder-7 (GAD-7) (17) The Pittsburgh Sleep Quality Index (PSQI) (18) and The Patient Health Questionnaire-9 (PHQ-9) questionnaires (19).

Outcomes measured included change in PHQ-9, GAD-7, PSQI, and QOL from baseline. The patients were asked to answer above mentioned questionnaires twice; initially, prior to their first session in treatment, and the second after at the end of study if they completed at least four cycle of medications. The changes in the answers were compared to determine the effectiveness of the Onabotulinumtoxin A treatment.

Improvement by ≥1 in severity category in the PHQ-9 or GAD-7 score from baseline was considered to be a clinically meaningful improvement (20, 21). On the other hand, a reduction by ≥3-point in the total PSQI score was considered a clinically meaningful improvement (22). Treatment satisfaction was assessed by numeric scale, in which 0 meant no satisfaction at all and 10 means the patient was fully satisfied.

This study was carried out in accordance with the ethical guidelines of Kuwait Ministry of Health. The protocol was approved by the ethical committee of Ibn Sina hospital. All subjects gave a written informed consent in accordance with the Declaration of Helsinki. The study was performed in observation of the latest version of the declaration of Helsinki (23), and all data was anonymous and protected in accordance with the ethical guidelines of the Council for International Organizations of Medical Sciences (24).

Statistical analyses were performed with IBM SPSS Statistics 25.0 software for Mac (SPSS Inc., Chicago, IL, USA). All continuous variables were expressed as means, whereas categorical ones were expressed as proportions and percentages. Paired sample *t*-test was used to compare between PHQ-9, GAD-7, PSQI, and QOL before and after treatment with Onabotulinumtoxin A. Pearson's correlations were performed for metric and ordinal variables between adverse events and quality of life. A significant difference was set to be at *p* < 0.05.

## Results

This cohort study included 139 CM patients. The eligible patients were131. Table 1 shows demographic and clinical characteristics of our cohort. The participants had mean age of 44.92 years, mean disease duration 12.20 years and a mean number of sessions 8. At the end of the study, most of the patients showed a significant improvement in the quality of life (81%), GAD-7 (81%), PHQ9 (79%), and PSQ1 (76%).

**Table 1:** Demographic and clinical characteristics of chronic migraine patients (*N* = 131).

**Variable**	**Mean ± SD/No (%)**
Age	44.92 ± 9.47
Range	23–65
**Gender**	
• Female	116 (88.5)
• Male	15 (11.5)
Disease duration in years	12.20 ± 10.10
Number of sessions	7.58 ± 3.36
**Education level**	
• Less than 12 years education	7 (5.3)
• 12 years education	14 (9.9)
• More than 12 years education	11 (84.7)
**Improvement of psychological aspects with Onabotulinumtoxin A**	
• QOL	81 (61.8)
• GAD-7	81 (61.8)
• PHQ9	79 (60.3)
• PSQI	76 (58)
Treatment satisfaction	7.21 ± 2.21

*QOL, Quality of Life; GAD-7, Seven-item Generalized Anxiety Disorder; PHQ9, Patient Health Questionnaire; PSQI, The Pittsburgh Sleep Quality Index*.

**Figure 1:**
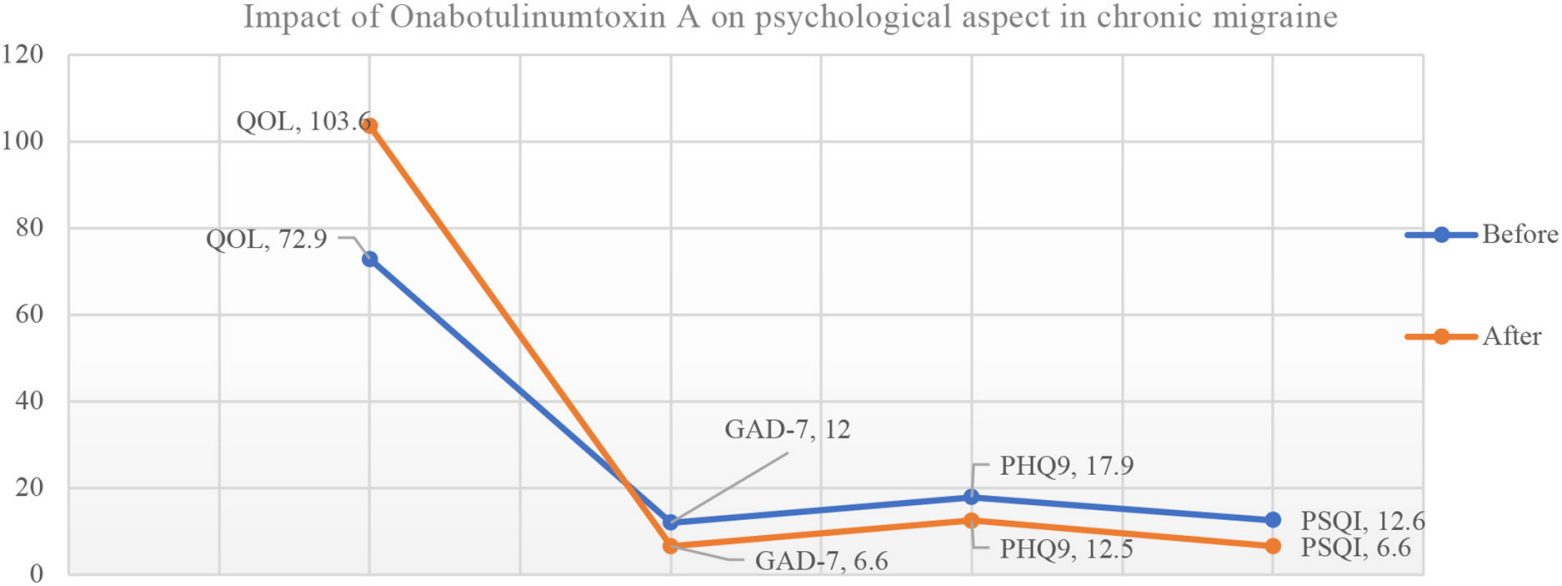
Impact of Onabotulinumtoxin A on psychological aspect in chronic migraine patients (*N* = 113). QOL, Quality of Life; GAD-7, Seven-item Generalized Anxiety Disorder; PHQ9, Patient Health Questionnaire; PSQI, The Pittsburgh Sleep Quality Index.

The mean score of patient satisfaction was 7.21. Onabotulinumtoxin A treatment for CM improved the quality of life significantly (72.92 vs. 103.62; *P* < 0.0001). It was also associated with significant reduction in the generalized anxiety scores (GAD-7) (12.0 vs. 6.6; *P* < 0.0001); depression scores (PHQ-9) (17.9 vs. 12.52; *P* < 0.0001) as well as sleep difficulty (PSQI) (12.6 vs. 6.7; *P* < 0.0001) at last visit (Table 2, Figure 1).

**Table 2:** Impact of Onabotulinumtoxin A on psychological aspect in chronic migraine patients (*N* = 113).

**Variables**	**Before treatment**	**After treatment**	***P*-Value**
	**Mean ± SD**	**Mean ± SD**	
QOL	72.92 ± 17.34	103.62 ± 15.62	0.0001*
GAD-7	12.00 ± 4.40	6.61 ± 4.40	0.0001*
PHQ9	17.91 ± 8.43	12.52 ± 8.77	0.0001*
PSQI	12.60 ± 5.66	6.66 ± 5.04	0.0001*

QOL, Quality of Life; GAD-7, Seven-item Generalized Anxiety Disorder; PHQ9, Patient Health Questionnaire; PSQI, The Pittsburgh Sleep Quality Index. *Statistically significant.

Our results did not conclude any significant difference among males and females regarding treatment satisfaction or improvement of psychological aspects (Table 3).

**Table 3:** Improvement of psychological aspects and treatment satisfaction with Onabotulinumtoxin A in both genders.

**Variables**	**Males (*N* = 15)**	**Females (*N* = 116)**	***P*-Value**
	***N* (%)**	***N* (%)**	
QOL	9 (60)	72 (62.1)	0.877
GAD-7	10 (66.7)	71 (61.2)	0.683
PHQ9	9 (60)	68 (58.6)	0.275
PSQI	11 (73.3)	67 (57.8)	0.869
Treatment satisfaction	7.87 ± 1.96	7.12 ± 2.24	0.221

*QOL, Quality of Life; GAD-7, Seven-item Generalized Anxiety Disorder; PHQ9, Patient Health Questionnaire; PSQI, The Pittsburgh Sleep Quality Index*.

We reported significant correlation between satisfaction for treatment and improvement of psychological aspects. However, we did not record any significant correlation between age, gender, education years, disease duration, education, and improvement of psychological aspects (Table 4). This means that improvement of psychological aspects in CM patients lead to treatment satisfaction regardless age, gender, disease duration, or education.

**Table 4:** Correlation between disease duration and treatment satisfaction or improvement of psychological aspects.

**Variables**	**Age**	**Gender**	**Disease duration**	**Education**	**Treatment satisfaction**
QOL	*R* = 0.007	*R* = 0.014	*R* = 0.003	*R* = 0.142	*R* = 0.294
	*P* < 0.383	*P* < 0.878	*P* < 0.9710	*P* < 0.106	*P* < 0.001*
GAD-7	*R* = 0.005	*R* = –0.036	*R* = −0.006	*R* = 0.172	*R* = 0.239
	*P* < 0.952	*P* < 0.685	*P* < 0.942	*P* < 0.049*	*P* < 0.006*
PHQ9	*R* = 0.066	*R* = −0.014	*R* = 0.42	*R* = 0.79	*R* = 0.195
	*P* < 0.453	*P* < 0.870	*P* < 0.635	*P* < 0.368	*P* < 0.0265*
PSQI	*R* = 0.034	*R* = −0.096	*R* = 0.058	*R* = 0.158	*R* = 0.239
	*P* < 0.699	*P* < 0.277	*P* < 0.508	*P* < 0.071	*P* < 0.006*

* *significant, QOL, Quality of Life; GAD-7, Seven-item Generalized Anxiety Disorder; PHQ9, Patient Health Questionnaire; PSQI, The Pittsburgh Sleep Quality Index*.

Few patients reported adverse effects related to Onabotulinumtoxin A treatment, which included neck pain and stiffness 3 (2.7%), lateral eyebrow elevation 2 (1.8%), ptosis 5 (4.44%). However, there was no significant correlation with these adverse events and quality of life after treatment (*P* < 0.62; *r* = 0.044). They were mild and irreversible.

## Discussion

Migraine is a common neurological disease that can be very disabling on the patients and their families. It is associated with reduced quality of life and other psychiatric comorbidities (25). Migraine is primarily managed through three approaches: lifestyle and behavioral modification, acute therapy, and prophylactic therapy to reduce the frequency, severity, and the duration of the attacks, and thus reducing the risk of medication overuse (26). The FDA has approved of Onabotulinumtoxin A for the prophylactic treatment of chronic migraine in 2010 (10). Its efficacy, safety and tolerability, were proven by the largest migraine therapeutic trial PREEMPT (27).

The analyses conducted in this study concluded that the majority of participants with chronic migraine were aged 45 years old on average, which is consistent with previous studies reporting that people in this age group are most likely to be affected by chronic migraine (28, 29). It was also noted that the majority of the participants were satisfied with their treatment. In addition, the study showed a significant improvement in the quality of life of patients with chronic migraine, which can be attributed to the successful migraine relief. This finding is consistent with previously reported studies (27, 30).

The mechanism by which Onabotulinumtoxin A decreases the burden of the disease is not entirely known, but it is theorized that Onabotulinumtoxin A works on the central, and peripheral sensitization and injecting the trigeminally innervated craniofacial cervical region will blocks the peripheral sensitization by inhibiting the release of pain mediating peptides, specifically CGRP (31).

Also, of note, anxiety and mood disorders have been shown to be closely associated with migraine (32, 33). It is documented that generalized anxiety disorder is 2–10 times more likely to affect migraineurs than the general population (32, 34). These psychiatric comorbidities may influence the prognosis of migraine as they have been associated with a poorer quality of life, increased suicide risk, and they are risk factors for progression of migraine from episodic to chronic (19, 32, 34). These results showed that Onabotulinumtoxin A used for the treatment of migraine helped with depression and anxiety in these patients, as there was a significant improvement in the PHQ-9 and GAD-7 questionnaires for depression and anxiety, respectively. This result was consistent with previous published studies (11, 35) that found that Onabotulinumtoxin A is associated with a reduction in the frequency and impact of migraine attacks. The studies also concluded that Onabotulinumtoxin A led to an improvement in the symptoms of depression and anxiety, for which the exact mechanism is not entirely clear yet. However, this may be attributed to either the placebo effect on headache and mood disturbances, or it can be due to the reduction of headaches, with a secondary reduction in depression and anxiety (35). On the other hand, there is some evidence that Onabotulinumtoxin A also can help in depression and anxiety independent of its effect on the improvement of migraine (36, 37). Therefore, using Onabotulinumtoxin A in patients with chronic migraine and other psychiatric comorbidities may be an excellent choice. Studies have also noted the cyclical relationship between migraines, stress, and anxiety, which can mean that the use of Onabotulinumtoxin A can lead to a decrease in migraine episodes both directly and indirectly through its effect of reducing anxiety and depression (38).

Moreover, previous Studies have shown a significant association between migraine and sleep disturbances (39, 40), as sleep disruption is common among migraine patients and can even trigger a migraine attack (41). The results of this study showed a significant reduction in the last visit compared to the first in the PSQI, which was employed to measure the quality and patterns of sleep. This is similar to the finding in a previous cross-sectional case control study that found PSQI scores increased with the increase in the frequency of migraine (41).

Satisfaction for treatment which reflects treatment-related migraine improvement correlated significantly with improvement of the investigated psychological aspects.

Some of the participants reported some of the documented side effects resulting from Onabotulinumtoxin A treatment, which included neck pain and stiffness, lateral eyebrow elevation, ptosis, and these adverse events were mild and reversible. These side effects are well-documented in studies that looked to Onabotulinumtoxin A efficacy (28). However, the number of patients experiencing these side effects was minimal 10(8.8%), and these adverse events was mild and reversible and did not impact quality of life in our cohort.

## Conclusion

This cohort study concluded that Onabotulinumtoxin A improves not only migraine symptoms in chronic migraine patients, but it also leads to significant improvements in psychological aspects in chronic migraine patients, leading to improvements in the quality of life, anxiety symptoms, as well as sleep disturbances. We believe that treating migraine patients should be targeting all these aspects since they are definitely synergistic to each other, they are bidirectional and treating one will improve the other and vice versa. Onabotulinumtoxin A may has the ability to do so without the need to use pharmacological agents.

## Strengths

This is the first study to be conducted in Kuwait in order to study the association between Onabotulinumtoxin A use for treatment of chronic migraine and psychological aspects and symptoms in chronic migraine. Another strength is the appropriate sample size and prospective study for 3 years. Nevertheless, some limitations were identified.

## Limitations

The limitations of the study are as follows: (1) the sample was chosen from the patient list in Ibn-Sina hospital, which may not completely represent all chronic migraine patients in Kuwait; (2) in addition, the study researched symptoms of psychiatric illnesses, whereas future studies can be dedicated to studying the effect of Onabotulinumtoxin A on patients with specific diagnoses concluded by psychiatrists through applying Diagnostic and Statistical Manual of Mental Disorders (DSM-5); (3) the study did not include a placebo group; (4) finally, the study did not report on the use of medication prior to initiation of Onabotulinumtoxin A treatment.

## Data Availability Statement

The raw data supporting the conclusions of this article will be made available by the authors, without undue reservation.

## Ethics Statement

The studies involving human participants were reviewed and approved by the ethical committee of Ibn Sina hospital. The participants provided written informed consent to participate in this study.

## Author Contributions

JA-H designed the study and reviewed the manuscript. HK, OA, FB, SM, and SA performed data collection and drafted the manuscript. DY and RA reviewed the manuscript, performed data collection and drafted the manuscript. SF performed statistical analysis, drafted, criticized, and reviewed the manuscript. All authors read and approved the final manuscript.

## Conflict of Interest

The authors declare that the research was conducted in the absence of any commercial or financial relationships that could be construed as a potential conflict of interest.

## Abbreviations

CGRP: Calcitonin gene-related peptide
CM: Chronic migraine
DSM-5: Diagnostic and Statistical Manual of Mental Disorders
FDA: Food and Drug Administration
ICHD-III: International Classification of Headache Disorders III
MDD: Major depressive disorder
MOH: Medication over use headache
PHQ9: Patient Health Questionnaire
PREEMPT: Phase III Research Evaluating Migraine Prophylaxis Therapy Trail
QOL: Quality of Life
GAD-7: Seven-item Generalized Anxiety Disorder
PSQI: The Pittsburgh Sleep Quality Index.
